# A role for subchondral bone changes in the process of osteoarthritis; a micro-CT study of two canine models

**DOI:** 10.1186/1471-2474-9-20

**Published:** 2008-02-12

**Authors:** Yvonne H Sniekers, Femke Intema, Floris PJG Lafeber, Gerjo JVM van Osch, Johannes PTM van Leeuwen, Harrie Weinans, Simon C Mastbergen

**Affiliations:** 1 Department of Orthopaedics, Erasmus MC, University Medical Center, Rotterdam, The Netherlands; 2 Department of Internal Medicine, Erasmus MC, University Medical Center, Rotterdam, The Netherlands; 3 Rheumatology & Clinical Immunology, University Medical Center Utrecht, Utrecht, The Netherlands; 4 Department of Otorhinolaryngology, Erasmus MC, University Medical Center, Rotterdam, The Netherlands

## Abstract

**Background:**

This study evaluates changes in peri-articular bone in two canine models for osteoarthritis: the groove model and the anterior cruciate ligament transection (ACLT) model.

**Methods:**

Evaluation was performed at 10 and 20 weeks post-surgery and in addition a 3-weeks time point was studied for the groove model. Cartilage was analysed, and architecture of the subchondral plate and trabecular bone of epiphyses was quantified using micro-CT.

**Results:**

At 10 and 20 weeks cartilage histology and biochemistry demonstrated characteristic features of osteoarthritis in both models (very mild changes at 3 weeks). The groove model presented osteophytes only at 20 weeks, whereas the ACLT model showed osteophytes already at 10 weeks. Trabecular bone changes in the groove model were small and not consistent. This contrasts the ACLT model in which bone volume fraction was clearly reduced at 10 and 20 weeks (15–20%). However, changes in metaphyseal bone indicate unloading in the ACLT model, not in the groove model. For both models the subchondral plate thickness was strongly reduced (25–40%) and plate porosity was strongly increased (25–85%) at all time points studied.

**Conclusion:**

These findings show differential regulation of subchondral trabecular bone in the groove and ACLT model, with mild changes in the groove model and more severe changes in the ACLT model. In the ACLT model, part of these changes may be explained by unloading of the treated leg. In contrast, subchondral plate thinning and increased porosity were very consistent in both models, independent of loading conditions, indicating that this thinning is an early response in the osteoarthritis process.

## Background

Osteoarthritis (OA) is a degenerative joint disease, which causes pain and disability and is characterized by progressive damage of articular cartilage, changes in the underlying (subchondral) bone, and occasional mild synovial inflammation.

Increasing evidence suggests that subchondral bone plays an important role in the etiology of OA [1,2], but studies thus far do not provide a consistent view on this subject. Subchondral bone changes have been studied in both humans with OA and in animal models of OA. In human studies, an increase in trabecular bone volume fraction and trabecular thickness was found [3,4], as well as an increase in cortical subchondral plate thickness [3]. However, other studies found a lower bone volume fraction and trabecular thickness in patients with OA [5,6] or a decrease in stiffness [7,8]. Even within one patient, areas with high and low bone volume fraction have been reported, depending on the condition of the overlying cartilage [9]. A problem of the human studies is that mostly established (severe) OA is studied, and longitudinal data showing the changes from onset until full clinical osteoarthritic signs do not exist. A problem is that there are no objective criteria that indicate early OA with mild pre-clinical signs and therefore the design of longitudinal studies is difficult.

Several animal models have been developed to study osteoarthritis and changes in the subchondral bone. Some animal studies reported a decrease in bone volume fraction and trabecular thickness [10-13], whereas in other studies these parameters increased [14,15]. These differences may be explained by the type of model used and the time at which the measurements were performed. Some bone parameters may occur in two phases: an initial decrease followed by an increase [16].

A frequently used animal model of OA is anterior cruciate ligament transection (ACLT) in dogs. ACLT results in permanent instability of the knee joint, which is followed by osteoarthritic features [17]. The ACLT model has been used for in-vivo evaluation of several treatment strategies [18-21]. However, the instability remains present, and may counteract the possible beneficial effects of treatment.

For this reason, the canine groove model has been developed. In this canine model, surgically applied damage to the articular cartilage of the weight-bearing areas of the femoral condyles, not damaging the subchondral bone, is the trigger for development of OA features [22]. The model is distinctive in that the osteoarthritic trigger is not permanent and the degenerative changes are progressive and not just the expression of surgically applied chondral damage, while synovial inflammation diminishes over time [22-24].

In the current study, we report changes in the subchondral bone of the canine groove model and compare these with changes in the ACLT model. Because the cartilage damage induced in the groove model appeared less drastic than in the ACLT model, the groove model could be very useful to investigate the subtle relationship between bone and cartilage during the development of OA. Therefore, we studied the groove model also at a very early time point. Specifically we used micro-CT analyses to quantify subchondral trabecular bone volume and architecture, the subchondral plate thickness and porosity, and osteophytosis and related this to the changes in cartilage integrity.

## Methods

OA was induced according to the ACLT model [25] or the groove model [22]. For the ACLT model, knee joints were available from 10 and 20 weeks post-surgery (both n = 5). For the groove model, knees were available from 3, 10 and 20 weeks post-surgery (all n = 4). In short, the following procedures were followed:

### Animals

22 female beagle dogs, aged 1.5–3 years and weighing 10–15 kg, were obtained from the animal laboratory at the Utrecht University, the Netherlands. They were housed in pairs in pens, and were let out for at least 2 hours daily on a patio in large groups. They were fed a standard diet and had water *ad libitum*. Ethical approval was given by the Utrecht University Medical Ethical Committee for animal studies.

### Anaesthesia, surgery, and post-surgical treatment

Procedures were carried out as described before [22-24]. Surgery was carried out through a 2–2.5 cm medial incision close to the *ligamentum patellae *in the right knee. Care was taken to limit bleeding and soft tissue damage. After surgery, synovium, fasciae and skin were sutured. The left unoperated knee served as a control. During the first 3 days after surgery, the dogs received analgesics (Buprenorphine 0.01 mg/kg) and antibiotics (Amoxicyclin 400 mg/kg). Daily release on the patio started 2 days after surgery. At the end of the experiment, the dogs were killed with an intravenous injection of Euthesate. Both hind limbs were amputated and within 2 hours proximal tibias and distal femurs were isolated and cartilage samples were collected.

### Groove model

**Figure 1:**
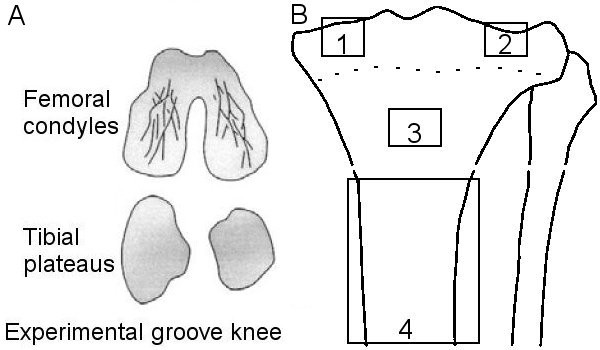
Schematic clarification of methods used. A: Localization of grooves made exclusively in the femoral condyles in the groove model. B: Selected regions that were analysed in the tibia using micro-CT. 1: cylinder in medial epiphysis; 2: cylinder in lateral epiphysis; 3: cylinder in metaphysis; 4: diaphysis. Cylinders 1 and 2 contain subchondral plate and trabecular bone. Cylinder 3 contains only trabecular bone. Region 4 contains only cortical bone. Dashed line indicates growth plate remnants.

In 12 animals, the cartilage of the lateral and medial femoral condyles was damaged with a Kirschner-wire (1.5 mm diameter) that was bent 90° at 0.5 mm from the tip as described before [22-24]. In this way the depth of the grooves was restricted to 0.5 mm. In utmost flexion, ten longitudinal and diagonal grooves were made on the weight-bearing parts of femoral condyles without damaging the subchondral bone (Fig. 1A). The latter was checked by histology at the end of the experiment. There was no absolute visual control over the procedure, but macroscopic evaluation after termination of the animals showed similar patterns in all knees treated. Two days after surgery, the dogs were forced to load the joint with the mechanically damaged cartilage by fixing the contra-lateral left limb to the trunk 3 days per week for approximately 4 h per day until the end of the experiment. The cartilage integrity and bone changes were evaluated 3, 10, and 20 weeks post-surgery (n = 4 in each group).

### ACLT model

In 10 animals, anterior cruciate ligament transection (ACLT) was carried out according to standard procedures using blunt curved scissors [25]. A positive anterior drawer sign confirmed completeness of the transection. The cartilage integrity and bone changes were evaluated 10 and 20 weeks post-surgery (n = 5 in each group).

### Cartilage integrity analysis

Cartilage integrity was evaluated both histochemically and biochemically [22-24]. Cartilage samples were obtained from predetermined locations on the weight-bearing areas of the femoral condyles and the tibial plateau of both experimental and control joints [22]. Cartilage was cut as thick as possible, while excluding the underlying bone (confirmed by histochemistry) and subsequently samples were cut into full-thickness cubes, weighing 3–10 mg (accuracy 0.1 mg).

For histology, 4 samples from tibial plateau and 4 from femoral condyles from each knee were fixed in 4% phosphate-buffered formalin containing 2% sucrose (pH 7.0). Cartilage degeneration was evaluated in safranin-O-fast-green iron hematoxylin-stained sections by light microscopy using the slightly modified [26] criteria of Mankin [27]. Specimens were graded in random order by two observers unaware of the source of the cartilage. For assessing the overall grade, the scores of the four specimens from each knee surface and of the two observers were averaged (a maximum of 11). This score of each joint surface was used as a representative score.

For femoral condyles and tibial plateau, the amount of GAG was determined as a measure of proteoglycan (PG) content of the cartilage. Six explants were taken from the experimental joint at fixed locations, which were paired with identical locations at the contralateral control joint. All samples were handled individually. The amount of GAG was determined as described previously [28]. Alcian blue staining of the medium was quantified photometrically with chondroitin sulphate (Sigma C4384) as a reference. Values were normalized to the wet weight of the cartilage explants (mg/g). The average result of the six samples was taken as representative of that joint surface [25].

### Micro-CT analysis

The proximal part of the tibias and the distal part of the femurs were scanned in a micro-CT scanner (Skyscan 1076, Skyscan, Antwerp, Belgium) with isotropic voxel size of 18 μm. The x-ray tube voltage was 60 kV and the current was 170 μA, with a 0.5 mm aluminium filter. The exposure time was 1180 ms. X-ray projections were obtained at 0.75° intervals with a scanning angular rotation of 198°. The reconstructed data set was segmented with a local thresholding algorithm [29]. The presence or absence of osteophytes in the reconstructed dataset was scored.

In both the medial and the lateral part of each femoral scan, a cylinder (diameter: 5.5 mm, height: 4.9 mm) was selected. Similarly, in the tibial scan, cylinders were selected with a diameter of 4.0 mm and a height of 3.5 mm (medial) or 3.1 mm (lateral) (Fig. 1B, regions 1 and 2). The cylinders were located in the middle of the load-bearing areas using anatomical landmarks. They contained trabecular bone and subchondral plate, but did not contain growth-plate tissue.

The trabecular bone and subchondral plate were separated automatically using in-house software. For the trabecular bone, bone volume fraction, which describes the ratio of bone volume over tissue volume (BV/TV), three-dimensional thickness (TbTh) [30], structure model index (SMI), a quantification of how rod-like or plate-like the bone structure is [31], and connectivity density (CD), describing the number of connections per volume [32], were calculated. For the subchondral plate, the three-dimensional thickness (PlTh) [30] and porosity (PlPor), describing the ratio of the volume of the pores in the plate over the total volume of the plate, were calculated. For these bone parameters, the data from the lateral and medial epiphyseal cylinders were averaged.

The potential effect of disuse of the joints due to the treatment procedures and/or the process of OA was investigated by analysing additional regions, further away from the joint space. A cylinder (width: 5.5 mm, height: 3.5 mm) was selected in the metaphysis of the tibia (Fig 1B, region 3), containing only trabecular bone, of which bone volume was calculated. Additionally, more distal in the tibia, a part of the diaphysis (height: 15.7 mm) was scanned at a resolution of 36 μm (Fig 1B, region 4). The diaphyseal scans were segmented with the same thresholding algorithm as the epiphyseal scans. The bone area and the corresponding moment of inertia (a parameter that reflects the distribution of the bone in each cross section) were calculated in the entire region, which contained predominantly cortical bone.

### Data analysis

The data are presented as absolute values, and as percentage difference or absolute difference of the experimental joint relative to the control joint. Since the sample sizes are small, a non-parametric paired test, the Wilcoxon signed rank test, was used to compare data for experimental and control joints. The cartilage parameters have been examined in previous studies with the same models [22,24], therefore we know the direction of the effect of these parameters. Thus for cartilage parameters one-sided p values are given. Since the bone parameters have never been studied in the groove model, we didn't know in advance in which direction the changes would evolve. Therefore two-sided p values are given for the bone parameters.

## Results

Changes in cartilage and in bone were similar for femoral condyles and tibial plateau. But for reasons of clarity the tibial plateau is shown as representative for both cartilage and bone parameters, since this surface was not surgically damaged when osteoarthritis was induced in the groove model, making comparison with the ACLT model the most sound.

### Groove vs. ACLT at 10 and 20 weeks post-surgery

#### Cartilage integrity

**Figure 2:**
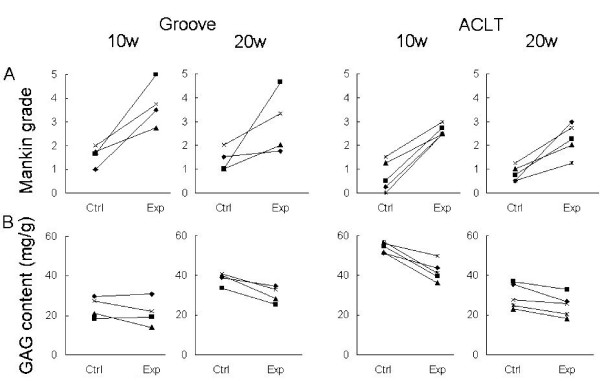
Cartilage integrity markers for individual animals. Data are shown for the tibial plateau of the groove and ACLT model at 10 and 20 weeks post-surgery. A: Mankin grade. B: GAG content.

Histological cartilage damage was increased in the experimental tibias of all animals in both models. (Table 1 and Fig 2A). This cartilage degradation was supported by biochemical analysis. A decrease in GAG content, representing impaired cartilage integrity, was found in the tibial plateau cartilage of both models. The GAG content was decreased with 10–25% in the experimental knee compared to the control knee (Table 1 and Fig 2B).

**Table 1:** Cartilage and bone parameters of the tibial epiphysis. Data are displayed as mean percentage difference (δ) or absolute difference (for Mankin grade and SMI) of the experimental OA joint relative to the contralateral control joint, for the groove model and ACLT model, at 3, 10, and 20 weeks post-surgery.

	**Cartilage**				**Epiphyseal trabecular bone**								**Metaphysis**		**Subchondral plate**			
	**GAG**		**Mankin**		**BV/TV**		**TbTh**		**SMI**		**CD**		**BV**		**PlTh**		**PlPor**	
	δ(%)	p	δ(-)	p	δ(%)	p	δ(%)	p	δ(-)	p	δ(%)	p	δ(%)	p	δ(%)	p	δ(%)	p
**Groove**																		
**3w**	-4.5	0.137	+0.17	0.055	+3.9	0.465	0.0	1.000	-0.05	0.465	+3.8	0.068	-0.9	0.715	-40.7	0.068	+84.8	0.068
**10w**	-11.1	0.233	+2.15	0.034	+6.0	0.068	+4.2	0.144	-0.30	0.068	+0.3	1.00	-3.1	0.068	-28.6	0.068	+47.7	0.068
**20w**	-20.9	0.034	+1.56	0.034	-3.5	0.144	-4.2	0.144	+0.28	0.068	+15.3	0.068	-12.5	0.144	-35.7	0.068	+72.2	0.068
**ACLT**																		
**10w**	-22.3	0.022	+1.95	0.021	-16.6	0.043	-12.2	0.043	+0.67	0.043	+20.9	0.225	-28.1	0.042	-28.7	0.043	+37.5	0.043
**20w**	-16.5	0.022	+1.45	0.021	-17.2	0.043	-13.6	0.043	+0.77	0.043	+19.5	0.043	-16.0	0.043	-30.9	0.043	+26.2	0.043

#### Osteophytes

**Figure 3:**
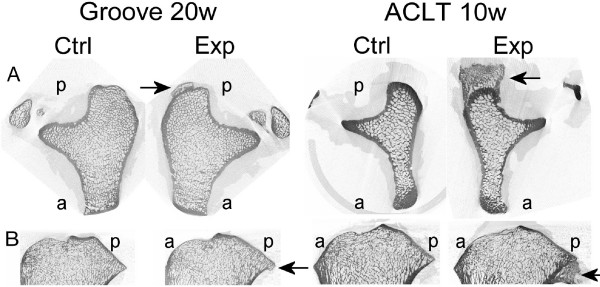
Osteophytes. A: Cross-sections of control and experimental tibia of groove at 20 weeks and ACLT at 10 weeks. Arrows indicate osteophytes; a = anterior, p = posterior. B: Longitudinal sections of tibias in A. Arrows indicate osteophytes; a = anterior, p = posterior.

In the groove model at 10 weeks post-surgery *no *osteophytes were found whereas at 20 weeks post-surgery they were clearly seen at the micro-CT images of the experimental tibial plateau in all four animals (Fig 3). In the ACLT model already at 10 weeks and also at 20 weeks post-surgery osteophytes were found at the experimental joint in all animals. For both models, the osteophytes were located predominantly at the medial site, below the rim of the tibia plateau. The osteophytes in the groove model were smaller than in the ACLT model. In none of the control joints osteophytes were observed.

#### Bone changes

##### Subchondral trabecular bone

**Figure 4:**
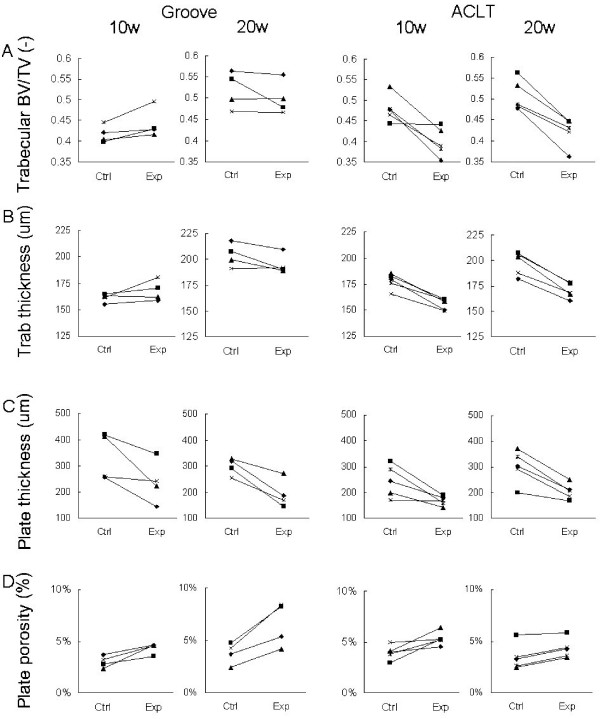
Bone parameters for individual animals. Data are shown for the tibial epiphysis of the groove and ACLT model at 10 and 20 weeks post-surgery. A: Trabecular bone volume fraction. B: Trabecular thickness. C: Subchondral plate thickness. D: Subchondral plate porosity.

Overall, the trabecular bone changes in the epiphysis of the experimental groove knee compared to its contralateral control were small. At 10 weeks there was a small increase in the trabecular bone volume fraction in the groove model. Also trabecular thickness was slightly elevated at 10 weeks. At 20 weeks the bone volume fraction and the trabecular thickness were slightly decreased (Table 1 and Fig. 4A, B).

The subchondral trabecular bone in the ACLT model showed a decrease in bone volume fraction (BV/TV) and trabecular bone thickness (TbTh) in all animals, at 10 and at 20 weeks. This was also reflected in the increase of the Structure Model Index (SMI) and Connectivity Density (CD) that indicate a more rod-like structure by the generation of more pores in the original structure, see Table 1.

##### Metaphyseal trabecular bone

In the metaphyseal region (region 3 in Fig 1B), which contained only trabecular bone, the differences in bone volume between control and experimental knee in the groove model were small. In the experimental ACLT knee, the bone volume was decreased up to 28% at 10 weeks (Table 1).

##### Subchondral plate

In contrast to the trabecular bone parameters, the changes in the subchondral plate were similar in both models. The thickness of the subchondral plate in the cylinders decreased in all animals with about 25 to 40% in both the groove and ACLT model at both time points. The porosity of the subchondral plate increased severely in both ACLT and groove model, at all time points in all animals (Table 1 and Fig 4C, D).

##### Diaphyseal cortical bone

In the diaphyseal part of the tibias, more distal from the joint, there were no differences in bone area and moment of inertia between the control knee and the experimental knee (data not shown) for both models.

### Groove model at 3 weeks post-surgery

The development of OA appeared less advanced in the groove model than in the ACLT model. Therefore we used the groove model to gain further insight in the subtle relationship between cartilage and bone in the process of OA development. Thus, an additional time point was studied, at 3 weeks post-surgery.

#### Cartilage integrity

**Figure 5:**
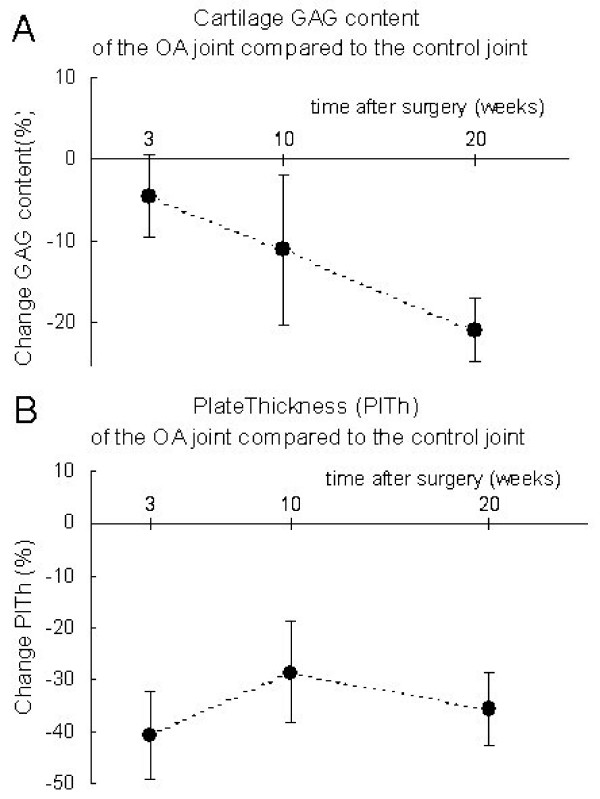
Relative change of experimental joints compared to control joints. Data are shown for the tibial epiphysis of the groove model at 3, 10, and 20 weeks post-surgery. A: Cartilage GAG content. B: Subchondral plate thickness. Error bars represent SEM.

The histological cartilage damage in the experimental tibia was minimal at 3 weeks, while at 10 and 20 weeks, more cartilage damage was present and in all animals. The GAG content was minimally reduced at 3 weeks and gradually decreased over time (Table 1 and Fig 5A).

#### Bone changes and osteophytes

##### Subchondral trabecular bone

At 3 weeks, there were no consistent changes in trabecular bone. Also in the metaphyseal area, no changes in trabecular bone were observed between experimental and control tibia.

##### Subchondral plate

In contrast to the trabecular bone, there were already clear changes in the subchondral plate at 3 weeks in the groove model. In all animals the subchondral plate thickness was decreased, on average with 40%. The plate porosity was increased in all animals, with on average 85%, which is even larger than at the later time points (Table 1 and Fig 5B).

No diaphyseal cortical bone changes or any osteophytes were found at 3 weeks post-surgery in the groove model.

## Discussion

The thickness of the subchondral plate decreased very consistently in two different canine models of osteoarthritis: the groove model and the ACLT model. In contrast, the changes in the trabecular bone at the tibial epiphysis in the groove model were relatively small and not consistent over time whereas these changes in the ACLT model were larger, with up to 20% loss in bone volume fraction with significant changes in the corresponding architectural parameters. Due to the low number of animals in the groove model, the bone parameters could not reach statistical significance in this model. Although the trabecular parameters were not consistent, the changes in the subchondral plate were very consistent in the groove model, with a clear and early reduction of the plate thickness and an increase in plate porosity.

Although the grooves in the groove model were made in the femur only, the changes in subchondral bone were found in both the femur and in the tibia. This is in concurrence with the cartilage changes found in the groove model which also showed changes in both femur and tibia [22]. Since the subchondral bone changes in the tibia cannot be caused directly by the grooves, we believe that these changes are part of the osteoarthritic process. This suggests an interaction between the bone and the cartilage through diffusive molecules that originate from the degenerated cartilage or the synovial fluid.

The cartilage changes in both models were similar to the changes previously described for larger groups of animals [22,24] and thus the data concerns a representative set of these earlier studies. The groove model showed only very mild changes in cartilage integrity at 3 weeks, which progressed at 10 and 20 weeks. In the ACLT model the changes were comparable to those in the groove model, but slightly more progressive.

Osteophytosis, visible on the CT-images, occurred in all the experimental ACLT knees at 10 and 20 weeks. This contrasts the groove model in which osteophytes only were detected at 20 weeks and not at 3 and 10 weeks. This corroborates the less progressive development of OA in the groove model compared to the ACLT model. However, a cartilaginous pre-form of the osteophytes may develop earlier, but is not detectable on the micro-CT images. In both models the osteophytes start below the rim of the medial tibia plateau and extend to more distant regions. This location is in line with osteophyte location in a rabbit ACLT model [16]. In human osteoarthritis, osteophytes are found close to the joint surface; it has been suggested that the load bearing area increases as to compensate for instability [33]. However, in our study, the osteophytes were also found in the groove model, in which the joint does not become unstable arguing against their role in joint stabilization. An explanation for the different location in comparison to humans may be that, in dogs, the ligaments are attached to the bone at a different location than in humans, thereby causing high stresses on the bone in a different location. In addition to this, cytokines such as TGFβ, which is elevated after OA induction [34,35], stimulate osteophyte formation [36]. Since the synovial capsule in dogs extends more to the proximal and distal part of the joint than in humans, the interface between synovial capsule and bone is more distant from the joint space. Assuming synovial tissue derived cytokines to play an important role in osteophyte formation [37], this may explain their location in dogs compared to humans.

The changes in the trabecular bone were not very pronounced in the groove model. However, in the ACLT model, the bone volume fraction and trabecular thickness were clearly reduced. This corroborates the difference in rate of development of cartilage changes in both models. The changes in the ACLT model fit with previous studies in this model in dogs as well as cats [10-13]. The fact that other studies find an increase in bone volume fraction and trabecular thickness [14,15] may be explained by the use of a different type of model, evaluated at a longer time period. Irrespective of the different changes in trabecular bone, similar changes in cartilage and subchondral plate were found in both models. Thus, it seems that the trabecular bone changes are not directly related to the changes in subchondral plate and cartilage. Since the subchondral plate changes consistently follow the cartilage changes, and the trabecular bone changes do not, the subchondral plate may play a more important role in the OA process than the trabecular bone changes.

The subchondral plate thickness decreased in both models at all time points in all experimental knees. This is in line with findings from previous studies concerning various animal models for OA, where subchondral plate thinning was documented in the early stage of the disease [10,11,38,39]. In some of these studies, this early phase of thinning was followed by a later phase of plate thickening [11,38]. This also explains the discrepancy with the sclerosis seen in most human studies [3,4,9], since such studies often concern patients with late osteoarthritis, whereas our present study examined only relatively early time points.

In order to justify the use of the contralateral knee as control, we calculated bone parameters in the diaphyseal and metaphyseal tibia, distal from the joint, containing cortical and trabecular bone, respectively. The bone volume of the metaphyseal tibia was significantly decreased in the experimental ACLT tibias, indicating disuse of the experimental ACLT knee. Thus, the trabecular bone loss in the epiphysis in the ACLT model may be explained by disuse. However, the tibias of the groove model showed hardly any changes in the diaphyseal and metaphyseal bone parameters. Hence, we have no signs of disuse in this model. Both the ACLT and groove model show similar subchondral plate thinning and increased porosity. Since the diaphyseal cortical bone showed no differences between control and experimental knee, we assume that in both models these subchondral plate changes are not caused by disuse of the treated leg.

The consistent decrease in subchondral plate thickness occurred already at 3 weeks post-surgery in the groove model, whereas the cartilage changes were only very mild at this early time point (Fig 5, table 1). This suggests that the subchondral plate changes occur fast. Taken together with the fact that this cannot be explained by disuse, this indicates (at least in the groove model) an interaction between cartilage and subchondral plate that induces bone resorption as a consequence of initiation of cartilage damage induced by the grooves. The thinning and drastically increased porosity of the subchondral plate may facilitate vascular invasion of the cartilage and diffusion of molecules from the damaged cartilage through the subchondral plate and vice versa, thereby enhancing the biochemical communication between bone and cartilage [40]. It is not clear if this bone cartilage communication interacts with an intrinsic repair activity of cartilage [41] or plays a role in the progression of the disease process [42].

## Conclusion

We see differences in subchondral trabecular bone changes and osteophyte formation between the groove model and the ACLT model, with the groove model clearly showing a slower development of these changes. However, the severe loss of thickness and increased porosity in the subchondral plate are the same in both models. This quick and extensive loss of the subchondral plate thickness and increase in plate porosity cannot be explained by unloading and strongly suggests that cartilage-bone interplay is part of the etiology of osteoarthritis.

## Abbreviations

ACLT: anterior cruciate ligament transection; Micro-CT, micro-computed tomography; OA, osteoarthritis; GAG, glycosaminoglycans; PG, proteoglycan; BV/TV, bone volume fraction; TbTh, trabecular thickness; SMI, structure model index; CD, connectivity density; PlTh, plate thickness; PlPor, plate porosity; TGFβ, transforming growth factor beta

## Competing interests

The author(s) declare that they have no competing interests.

## Authors' contributions

YS carried out the bone analysis and drafted the manuscript. FI carried out the cartilage analysis. FL and SM designed the study. All authors were involved in interpretation of the data and revision of the manuscript. All authors read and approved the final manuscript.

## Pre-publication history

The pre-publication history for this paper can be accessed here:



## References

[B1] Radin EL, Rose RM (1986). Role of subchondral bone in the initiation and progression of cartilage damage. Clin Orthop.

[B2] Burr DB (1998). The importance of subchondral bone in osteoarthrosis. Curr Opin Rheumatol.

[B3] Grynpas MD, Alpert B, Katz I, Lieberman I, Pritzker KP (1991). Subchondral bone in osteoarthritis. Calcif Tissue Int.

[B4] Bobinac D, Spanjol J, Zoricic S, Maric I (2003). Changes in articular cartilage and subchondral bone histomorphometry in osteoarthritic knee joints in humans. Bone.

[B5] Patel V, Issever AS, Burghardt A, Laib A, Ries M, Majumdar S (2003). MicroCT evaluation of normal and osteoarthritic bone structure in human knee specimens. J Orthop Res.

[B6] Messent EA, Ward RJ, Tonkin CJ, Buckland-Wright C (2007). Osteophytes, juxta-articular radiolucencies and cancellous bone changes in the proximal tibia of patients with knee osteoarthritis. Osteoarthritis Cartilage.

[B7] Li B, Aspden RM (1997). Mechanical and material properties of the subchondral bone plate from the femoral head of patients with osteoarthritis or osteoporosis. Ann Rheum Dis.

[B8] Day JS, Ding M, van der Linden JC, Hvid I, Sumner DR, Weinans H (2001). A decreased subchondral trabecular bone tissue elastic modulus is associated with pre-arthritic cartilage damage. J Orthop Res.

[B9] Chappard C, Peyrin F, Bonnassie A, Lemineur G, Brunet-Imbault B, Lespessailles E, Benhamou CL (2006). Subchondral bone micro-architectural alterations in osteoarthritis: a synchrotron micro-computed tomography study. Osteoarthritis Cartilage.

[B10] Pelletier JP, Boileau C, Brunet J, Boily M, Lajeunesse D, Reboul P, Laufer S, Martel-Pelletier J (2004). The inhibition of subchondral bone resorption in the early phase of experimental dog osteoarthritis by licofelone is associated with a reduction in the synthesis of MMP-13 and cathepsin K. Bone.

[B11] Dedrick DK, Goldstein SA, Brandt KD, O'Connor BL, Goulet RW, Albrecht M (1993). A longitudinal study of subchondral plate and trabecular bone in cruciate-deficient dogs with osteoarthritis followed up for 54 months. Arthritis Rheum.

[B12] Boyd SK, Muller R, Leonard T, Herzog W (2005). Long-term periarticular bone adaptation in a feline knee injury model for post-traumatic experimental osteoarthritis. Osteoarthritis Cartilage.

[B13] Boyd SK, Muller R, Matyas JR, Wohl GR, Zernicke RF (2000). Early morphometric and anisotropic change in periarticular cancellous bone in a model of experimental knee osteoarthritis quantified using microcomputed tomography. Clin Biomech (Bristol, Avon).

[B14] Layton MW, Goldstein SA, Goulet RW, Feldkamp LA, Kubinski DJ, Bole GG (1988). Examination of subchondral bone architecture in experimental osteoarthritis by microscopic computed axial tomography. Arthritis Rheum.

[B15] Ding M, Danielsen CC, Hvid I (2006). Age-related three-dimensional microarchitectural adaptations of subchondral bone tissues in guinea pig primary osteoarthrosis. Calcif Tissue Int.

[B16] Batiste DL, Kirkley A, Laverty S, Thain LM, Spouge AR, Holdsworth DW (2004). Ex vivo characterization of articular cartilage and bone lesions in a rabbit ACL transection model of osteoarthritis using MRI and micro-CT. Osteoarthritis Cartilage.

[B17] Brandt KD, Myers SL, Burr D, Albrecht M (1991). Osteoarthritic changes in canine articular cartilage, subchondral bone, and synovium fifty-four months after transection of the anterior cruciate ligament. Arthritis Rheum.

[B18] Caron JP, Fernandes JC, Martel-Pelletier J, Tardif G, Mineau F, Geng C, Pelletier JP (1996). Chondroprotective effect of intraarticular injections of interleukin-1 receptor antagonist in experimental osteoarthritis. Suppression of collagenase-1 expression. Arthritis Rheum.

[B19] Manicourt DH, Altman RD, Williams JM, Devogelaer JP, Druetz-Van Egeren A, Lenz ME, Pietryla D, Thonar EJ (1999). Treatment with calcitonin suppresses the responses of bone, cartilage, and synovium in the early stages of canine experimental osteoarthritis and significantly reduces the severity of the cartilage lesions. Arthritis Rheum.

[B20] Pelletier JP, Lascau-Coman V, Jovanovic D, Fernandes JC, Manning P, Connor JR, Currie MG, Martel-Pelletier J (1999). Selective inhibition of inducible nitric oxide synthase in experimental osteoarthritis is associated with reduction in tissue levels of catabolic factors. J Rheumatol.

[B21] Yu LP, Smith GN, Brandt KD, Myers SL, O'Connor BL, Brandt DA (1992). Reduction of the severity of canine osteoarthritis by prophylactic treatment with oral doxycycline. Arthritis Rheum.

[B22] Marijnissen AC, van Roermund PM, TeKoppele JM, Bijlsma JW, Lafeber FP (2002). The canine 'groove' model, compared with the ACLT model of osteoarthritis. Osteoarthritis Cartilage.

[B23] Marijnissen AC, van Roermund PM, Verzijl N, Tekoppele JM, Bijlsma JW, Lafeber FP (2002). Steady progression of osteoarthritic features in the canine groove model. Osteoarthritis Cartilage.

[B24] Mastbergen SC, Marijnissen AC, Vianen ME, van Roermund PM, Bijlsma JW, Lafeber FP (2006). The canine 'groove' model of osteoarthritis is more than simply the expression of surgically applied damage. Osteoarthritis Cartilage.

[B25] van Valburg AA, van Roermund PM, Marijnissen AC, Wenting MJ, Verbout AJ, Lafeber FP, Bijlsma JW (2000). Joint distraction in treatment of osteoarthritis (II): effects on cartilage in a canine model. Osteoarthritis Cartilage.

[B26] Lafeber FP, Vander Kraan PM, Huber-Bruning O, Vanden Berg WB, Bijlsma JW (1993). Osteoarthritic human cartilage is more sensitive to transforming growth factor beta than is normal cartilage. Br J Rheumatol.

[B27] Mankin HJ, Dorfman H, Lippiello L, Zarins A (1971). Biochemical and metabolic abnormalities in articular cartilage from osteo-arthritic human hips. II. Correlation of morphology with biochemical and metabolic data. J Bone Joint Surg Am.

[B28] Lafeber FP, Vander Kraan PM, Van Roy JL, Huber-Bruning O, Bijlsma JW (1993). Articular cartilage explant culture; an appropriate in vitro system to compare osteoarthritic and normal human cartilage. Connect Tissue Res.

[B29] Waarsing JH, Day JS, Weinans H (2004). An improved segmentation method for in-vivo micro-CT imaging. J Bone Miner Res.

[B30] Hildebrand T, Ruegsegger P (1997). A new method for the model-independent assessment of thickness in three-dimensional images. J Micros.

[B31] Hildebrand T, Ruegsegger P (1997). Quantification of Bone Microarchitecture with the Structure Model Index. Comput Methods Biomech Biomed Engin.

[B32] Odgaard A, Gundersen HJ (1993). Quantification of connectivity in cancellous bone, with special emphasis on 3-D reconstructions. Bone.

[B33] Dayal N, Chang A, Dunlop D, Hayes K, Chang R, Cahue S, Song J, Torres L, Sharma L (2005). The natural history of anteroposterior laxity and its role in knee osteoarthritis progression. Arthritis Rheum.

[B34] Fahlgren A, Andersson B, Messner K (2001). TGF-beta1 as a prognostic factor in the process of early osteoarthrosis in the rabbit knee. Osteoarthritis Cartilage.

[B35] Hayami T, Pickarski M, Wesolowski GA, McLane J, Bone A, Destefano J, Rodan GA, Duong le T (2004). The role of subchondral bone remodeling in osteoarthritis: reduction of cartilage degeneration and prevention of osteophyte formation by alendronate in the rat anterior cruciate ligament transection model. Arthritis Rheum.

[B36] van Beuningen HM, van der Kraan PM, Arntz OJ, van den Berg WB (1994). Transforming growth factor-beta 1 stimulates articular chondrocyte proteoglycan synthesis and induces osteophyte formation in the murine knee joint. Lab Invest.

[B37] Blom AB, van Lent PL, Holthuysen AE, van der Kraan PM, Roth J, van Rooijen N, van den Berg WB (2004). Synovial lining macrophages mediate osteophyte formation during experimental osteoarthritis. Osteoarthritis Cartilage.

[B38] Hayami T, Pickarski M, Zhuo Y, Wesolowski GA, Rodan GA, Duong LT (2006). Characterization of articular cartilage and subchondral bone changes in the rat anterior cruciate ligament transection and meniscectomized models of osteoarthritis. Bone.

[B39] Botter SM, van Osch GJVM, Waarsing JH, Day JS, Verhaar JAN, Pols HAP, van leeuwen JPTM, Weinans H (2006). Quantification of subchondral bone changes in a murine osteoarthritis model using micro-CT. Biorheology.

[B40] Buckwalter JA, Mankin HJ (1997). Articular cartilage: degeneration and osteoarthrosis, repair, regeneration, and transplantation. J Bone Joint Surg Am.

[B41] Lafeber FP, van Roy H, Wilbrink B, Huber-Bruning O, Bijlsma JW (1992). Human osteoarthritic cartilage is synthetically more active but in culture less vital than normal cartilage. J Rheumatol.

[B42] Westacott CI, Webb GR, Warnock MG, Sims JV, Elson CJ (1997). Alteration of cartilage metabolism by cells from osteoarthritic bone. Arthritis Rheum.

